# Exploring the Impact of a Mobile Health Solution for Postpartum Pelvic Floor Muscle Training: Pilot Randomized Controlled Feasibility Study

**DOI:** 10.2196/12587

**Published:** 2019-07-11

**Authors:** Sinéad Dufour, Donna Fedorkow, Jessica Kun, Shirley Xiaoxuan Deng, Qiyin Fang

**Affiliations:** 1 School of Rehabilitation Science McMaster University Hamilton, ON Canada; 2 Department of Obstetrics & Gynecology McMaster University Hamilton, ON Canada; 3 School of Biomedical Engineering McMaster University Hamilton, ON Canada; 4 Department of Engineering Physics McMaster University Hamilton, ON Canada

**Keywords:** postpartum, pelvic floor, mobile health, feasibility study, wireless technology, wearable technology, computer games, biofeedback

## Abstract

**Background:**

The postpartum period is a vulnerable time for the pelvic floor. Early implementation of pelvic floor muscle exercises, appropriately termed as pelvic floor muscle training (PFMT), in the postpartum period has been advocated because of its established effectiveness. The popularity of mobile health (mHealth) devices highlights their perceived utility. The effectiveness of various mHealth technologies with claims to support pelvic floor health and fitness is yet to be substantiated through systematic inquiry.

**Objective:**

The aim of this study was to determine the acceptability, feasibility, and potential effect on outcomes of an mHealth device purposed to facilitate pelvic floor muscle training among postpartum women.

**Methods:**

A 16-week mixed methods pilot study was conducted to evaluate outcomes and determine aspects of acceptability and feasibility of an mHealth device. All participants received standardized examination of their pelvic floor muscles and associated instruction on the correct performance of PFMT. Those randomized to the iBall intervention received instructions on its use. Schedules for utilization of the iBall and PFMT were not prescribed, but all participants were informed of the standard established recommendation of PFMT, which includes 3 sets of 10 exercises, 3 to 4 times a week, for the duration of the intervention period. Quantitative data included the measurement of pelvic floor muscle parameters (strength, endurance, and coordination) following the PERFECT assessment scheme: Incontinence Impact Questionnaire scores and the Urogenital Distress Inventory (UDI-6) scores. Aspects of acceptability and feasibility were collected through one-to-one interviews. Interview transcripts were analyzed using Thorne’s interpretive description approach.

**Results:**

A total of 23 women with a mean age of 32.2 years were randomized to an intervention group (n=13) or a control group (n=10). Both groups improved on all measures. The only statistically significant change was the UDI-6 score within both groups at 16 weeks compared with baseline. There was no statistically significant difference between the intervention group and control group on any outcomes. Most participants using the iBall (n=10, 77%) indicated value in the concept of the mHealth solution. Technical difficulties (n=10, 77%), a cumbersome initiation process (n=8, 61%), and discomfort from the device (n=8, 61%) were reasons impeding intervention acceptability. Most participants (n=17, 74%) indicated that the initial assessment and training was more useful than the mHealth solution, a tenet that was echoed by all control group participants.

**Conclusions:**

Our pilot study demonstrated the potential for mHealth solution–enhanced PFMT in the early postpartum period. Usability issues in hardware and software hindered feasibility and acceptance by the participants. Our findings can inform the redesign of mHealth solutions that may be of value if acceptability and feasibility issues can be overcome.

**Trial Registration:**

ClinicalTrials.gov NCT02865954; https://clinicaltrials.gov/ct2/show/NCT02865954

## Introduction

The postpartum period is a vulnerable time for the pelvic floor [1]. Dysfunction of the pelvic floor musculature is associated with urinary and fecal incontinence, pelvic organ prolapse, and lumbopelvic pain [2-8]. Prevalence data vary; however, approximately 23% of postpartum women report either urinary or fecal incontinence [9].

Urinary incontinence represents the most prevalent issue and can be associated with reduced quality of life and mental health sequelae, such as depression and anxiety [10-14]. Stress urinary incontinence is defined as the involuntary loss of urine on effort or physical exertion, including coughing or sneezing [15]. It is associated with pregnancy, labor management, and birth and often manifests in the postpartum period [16-18].

Pelvic floor muscle training (PFMT) denotes individually tailored pelvic floor muscle rehabilitation programs aimed at restoring the fitness and function of the pelvic floor and associated deep core musculature. Early implementation of PFMT in the postpartum has been advocated based on 2 rigorously conducted reviews confirming prevention and correction of urinary incontinence through pelvic floor rehabilitation [19,20]. Research supports postpartum care to include basic evaluation of the pelvic floor musculature and associated education as well as feedback to ensure the correct performance of pelvic floor muscle exercises, namely PFMT [21,22]. PFMT can be effectively initiated with brief instruction through digital palpation of the pelvic floor muscles as associated feedback related to the muscle contraction completed [21]. It is established that, in the absence of providing adequate instruction and biofeedback, it is difficult for women to accurately perform pelvic floor muscle exercises [23,24]. Existing literature suggests that best practices for the prevention and management of pelvic floor dysfunction are not routinely implemented [25,26]. There is no universally accepted protocol for the initiation of PFMT in the postpartum period. This represents a research-practice gap in optimizing pelvic floor health in the postpartum period and beyond.

A proactive approach to pelvic health is needed as are strategies to overcome barriers contributing to this research-practice gap. Mobile health (mHealth) solutions may provide strategies to close the gap. mHealth solutions are defined by the World Health Organization [27] as the “medical and public health practice supported by mobile devices, such as mobile phones, patient monitoring devices, personal digital assistants, and other wireless devices” [27]. The current market for mHealth technologies is growing, including applications marketed for pelvic floor rehabilitation and fitness [28-31]. These mHealth technologies may represent innovative modalities to support pelvic floor muscle fitness. Claims and proposed benefits of these mHealth solutions, compared with standard care, are based on concepts and have not been substantiated by scientific investigation. Overall, the body of evidence on the effectiveness of mHealth technology is, in general, weak [32].

This pilot randomized controlled study aimed to evaluate the feasibility and acceptability of an mHealth solution (iBall) as a rehabilitation tool to support PFMT efforts in the early postpartum period. The iBall is a device that, upon insertion into the vaginal canal, can detect the strength and muscular endurance of pelvic floor muscles. The results are displayed in the accompanying mobile app that incorporates various training routines and gaming options. It aims to encourage both adherence to and improvement of outcomes of PFMT. Specifically, we sought to assess the feasibility, acceptability, and effectiveness of outcomes of an mHealth device (iBall) compared with PFMT instruction alone in the early postpartum period.

## Methods

### Sample and Recruitment

**Figure 1:**
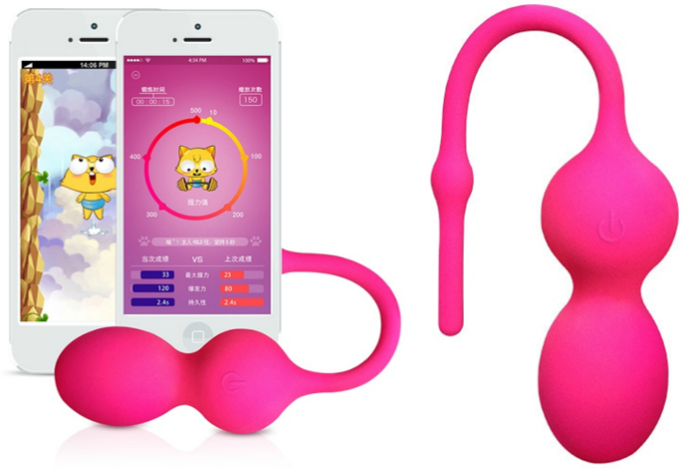
The mHealth iBall device. The iBall device consists of a Bluetooth antenna that sits outside of the body and 2 spherical compartments containing a battery and biofeedback sensor that sits within the vaginal canal.

Women in the third trimester of pregnancy, attending local midwifery practices, were invited to participate in the study. Posters were used to facilitate recruitment. All initial study procedures took place in the early postpartum period (within the fourth trimester, the first 13 weeks postpartum). Following initial screening and confirmation of eligibility, participants were prospectively randomized to an mHealth-assisted PFMT intervention or PFMT instruction only (Figure 1). The study was approved by the Hamilton Integrated Research and Ethics Board.

### Intervention

The iBall (ChunShuiTang Co Ltd) is an interactive mHealth device designed to facilitate PFMT. The iBall device is a US Federal Communications Commission–certified [33] and European Conformity–marked [34,35] device that, upon insertion into the vaginal canal, can detect the strength (power generation) and associated muscular endurance of pelvic floor muscles. Currently, iBall has been approved for consumer use in Europe and China. This information detected from the sensors within the device are transmitted via Bluetooth to a smartphone app that guides users in various exercise programs. The device is shown in Figure 1. It comprises 2 spherical compartments and a Bluetooth antenna for wireless communication with the smartphone. The whole device including the antenna is encapsulated in waterproof medical grade silicone casing. The battery and charging circuit are in the top spherical compartment with a waterproof charging port in the silicone encapsulation.

**Figure 2:**
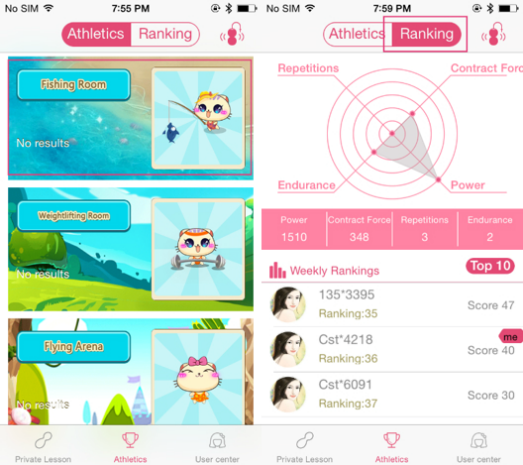
The iBall App. Left: There are a number of activities that aid in engaging the pelvic floor. Right: The progress of the user can be tracked and monitored.

The sensor and biofeedback motors are in the bottom compartment, which also contains a power button under the silicone casing. The user can press and hold the power button to turn on/off the device. The antenna also serves as a handle to push in and pull out the device from the vaginal canal. In this study, each participant in the intervention group will receive an iBall device along with an already paired smartphone (iPhone 5e) with the mobile app installed and tested with the device. Both the iBall device and the smartphone were originally fully charged, and power will last a few days depending on usage level. They both require periodic charging by the participants at home during the study period. Once inserted, the user has several interactive games or activities to choose from on the corresponding app on their smartphone (Figure 2). Their progress can also be tracked, both as a score in the game and as a measure of power, endurance, repetitions, and contracting force (Figure 2).

These quantitative results are saved with a time stamp in the mobile app. The data can also be automatically uploaded to an encrypted cloud storage server through an *opt in* option. In this study, the participants have consented to upload their usage data for the study with the uploading feature enabled. Each user’s study smartphone app has already been configured with a unique randomly generated user account and log-in credential. Only the research team has a securely saved lookup table linking the user account and the identity of the participants. The cloud-stored data can be downloaded as structured data files (in American Standard Code for Information Interchange format) for further analysis. The original consumer product has a Web-based profile function allowing the users to access their data on the cloud server and interact (by users’ choice) with other users in a Web-based community. This feature is only available in Chinese and was permanently disabled in the clinical study version of the smartphone app. As a result, although the device and app have the capability of Web access to their usage data and interacting with other users, such functionalities were disabled in devices used in this study. The iBall device is currently marked as a consumer electronics gaming device. Some quantitative results (eg, *power* or *contracting force*) are not calibrated for absolute pressure measurements. The results of metrics obtained from the devices are not included in this study.

### Research Design

This study was done in a 1:1 allocation ratio using random number assignments. Allocations were placed in sealed envelopes that were opened after the initial physical assessment at the time of randomization. Women having a vaginal or cesarean birth within 21 weeks of delivery were included. To maximize generalizability, the exclusions were made based on the individuals’ inability to understand and read English and direction from their caregivers to not insert anything into their vagina.

An initial phone screening was conducted by a research assistant. Interested and eligible participants then met with a research assistant for further discussion and to obtain informed consent. The start time of the intervention took place in the early postpartum period, primarily during the fourth trimester (from week 6 postpartum to week 13 postpartum). The baseline characteristics of the participants have been provided in Table 1.

Subjects were then assessed by one of the expert assessors (SD and DF). The assessment included completion of 2 self-report outcome measures and a digital pelvic floor examination, inclusive of initiating PFMT by one of the 2 assessors. The 2 validated self-report measures that were administered were the Urogenital Distress Inventory (UDI-6) [36] and the Incontinence Impact Questionnaire (IIQ-7), which are shown in Table 2. The pelvic floor muscle examination followed the PERFECT scheme [8]. Clinically relevant changes in the PERFECT score was predetermined as an improvement of 20% based on previously published minimal clinical improvement thresholds (Table 3) [37].

Following assessment, subjects received their randomization allocation. Subjects randomized to the iBall group received additional instruction and training from the research assistant. Follow-up assessments were completed by the same assessors (SD and DF). A consistent process for assessment procedures including cueing during the pelvic floor muscle testing and instruction for the performance of pelvic floor exercises was established. All participants received instruction for the correct performance of pelvic floor muscle exercises via digital palpation. Schedules for utilization of the iBall and PFMT were not prescribed, but all participants were informed of the standard established recommendation of PFMT, which includes 3 sets of 10 exercises, 3 to 4 times a week, for duration of the intervention period [38]. Neither assessor was at any time, during the conduct of the study, aware of which group a subject was allocated.

Participants randomized to the intervention group received additional instruction on how to use the mHealth solution (iBall) by a research assistant. Participants were shown how to use the device and were familiarized with the associated app on the associated smartphone. Maintenance was informed as per manufacturers recommendations, which involved washing the device with warm water and a natural soap and storing it in the case it came in. Participants then used the device according to their own schedule (eg, when to exercise, frequency, and how long). The PFMT with iBall occurs within the context of playing a variety of games. Participants in the intervention group received a *booster session*, midway through the intervention period. This booster session consisted of an email sent by a research assistant reminding participants of features of the *iBall* and benefits of postpartum pelvic floor muscle exercise as a means of self-management support. The use of booster sessions to facilitate self-management support has been shown to be a useful procedure in rehabilitation interventions [40]. Quantitative measures were obtained at baseline and following 16 weeks of treatment. The duration of treatment was chosen based on the existing literature using 12 or more weeks as a reasonable therapeutic window [41,42]. As this was a pilot study, a convenient sample size of 23 was selected. This size appeared reasonable, given other comparison studies of PFMT [43].

**Table 1:** Baseline characteristics and participant demographics.

Category			iBall, n (%)		Control, n (%)
**Age (years)**					
	26-30	5 (21.7)		1 (4.3)	
	31-36	5 (21.7)		5 (21.7)	
	Unknown	3 (13.0)		4 (17.4)	
**Number of deliveries**					
	1	6 (26.1)		1 (4.3)	
	2	6 (26.1)		6 (26.1)	
	3	0		1 (4.3)	
	4	1 (4.3)		0	
	Unknown	0		2 (8.7)	
**Weeks postpartum**					
	<7	2 (8.7)		2 (8.7)	
	7-14	10 (43.5)		7 (30.4)	
	>14	1 (4.3)		0	
**PERFECT^a^ score**					
	Power (<3/5)	2 (15.4)		4 (40)	
	Endurance (<6/10)	5 (38.5)		5 (50)	
	Repetition (<6/10)	7 (53.8)		7 (70)	
	Fast (<6/10)	6 (46.2)		6 (60)	
	Coordination (No)	6 (46.2)		6 (60)	
	Timing (No)	4 (30.8)		4 (40)	

^a^PERFECT is an acronym where P is power or pressure, E is endurance, R is repetitions, F is fast contractions, and ECT is every contraction timed. The scheme was developed to simplify and clarify pelvic floor muscle assessment [8].

**Table 2:** Baseline characteristics and participant demographics: urogenital distress inventory and Incontinence Impact Questionnaire scores.

Category	iBall		Control	
	Mean	Range (SD)	Mean	Range (SD)
Age (years)	31	26-34 (2.7)	34	29-36 (2.2)
UDI^a^-6	18.9	0-47 (11.5)	25.4	0-54 (15.9)
IIQ^b^-7	8.1	0-50 (15.6)	7.4	0-28 (11.4)

^a^UDI: Urogenital Distress Inventory.

^b^IIQ: Incontinence Impact Questionnaire.

**Table 3:** Operational definitions and psychometric properties of outcome measures.

Assessment, references, and description of procedure		Interpretation	Reported psychometric properties
**UDI^a^-6 [36]**			
	Designed to assess the degree to which symptoms associated with incontinence are troubling. The weight of accumulated evidence suggests that the both the UDI long and short forms are validated.	Symptoms scored according to self-rated severity.	Internal consistency=0.52; Correlation with long version=0.87
**IIQ^b^-7 [36]**			
	Designed to assess the impact of urinary incontinence on activities and emotions. The weight of accumulated evidence suggests that both the UDI long and short forms are validated.	Symptoms scored according to self-rated severity.	Internal consistency=0.84; Correlation with long version=0.95
**PERFECT score [8,39]**			
	P—power, using the Modified Oxford grading scale: 0-no contraction; 1-flicker; 2-weak squeeze, no lift; 3-fair squeeze, definite lift; 4-good squeeze with lift; 5-strong squeeze with a lift; Positive test: <4/5	Pelvic floor muscle strength	Kappa=0.48-0.77
	E—endurance, the time (in seconds) that a maximum contraction can be sustained; Positive test: <10 seconds	Pelvic floor muscle endurance	Kappa=0.17-0.56
	R—repetition, the number of repetitions of a maximum voluntary contraction; Positive test: <10 repetitions	Pelvic floor muscle endurance	Kappa=0.48-0.77
	F—fast contractions, the number of fast (1 second) maximum contractions; Positive test: <10 repetitions	Pelvic floor muscle responsiveness	Kappa=0.29-0.65
	ECT—timing-sustained voluntary contraction of the pelvic floor muscles with a cough; Positive test: no contraction of pelvic floor muscles before cough [35]	Pelvic floor muscle coordination	Kappa=0.14-0.53

^a^UDI: Urogenital Distress Inventory.

^b^IIQ: Incontinence Impact Questionnaire.

All quantitative data were inputted into an Excel spreadsheet (version 2017) for analysis. Data were cleaned and checked for out-of-range values, skip pattern problems, and duplicates. Tests of normality were completed to determine appropriateness of statistical methods. Analyses were performed using an intention-to-treat basis. All statistical tests used 2-sided tests at the .05 level of significance. Descriptive analyses of participants’ characteristics and feasibility of the intervention were expressed as a mean (standard deviation) or median (minimum-maximum) for continuous variables and count (percent) for categorical variables. Changes in outcomes over time were examined using paired *t* tests for continuous variables and Fischer exact test for categorical variables.

In addition to repeating all baseline assessments, the postintervention assessments required additional data collection from both groups to determine the implementation outcomes of acceptability and feasibility of the intervention (Table 4). The PFMT instruction only (control) group answered 3 self-administered questions.

1. Are you having any problems that you attribute to your pelvic floor needing rehabilitation (urinary or fecal incontinence, pelvic or lower back pain, painful intercourse, or pelvic pressure)?
2. Consider the last few months since your baseline assessment, is there anything that you think might have been useful to enhance your physical postpartum recovery?
3. If you had the opportunity to use a mobile health app designed to assist with PFMT (eg, iBall), would you want to try it?

Aspects of acceptability and feasibility were collected through one-to-one interviews. Interviews were conducted by one of the 2 study assessors (SD and SD); transcripts were analyzed using Thorne’s interpretive description approach [44]. The larger purpose of this study was to seek an in-depth understanding of the mHealth solution studied, such as to inform clinical practice. This included assessment of the interventions’ perceived effects, barriers, and facilitators to implementation and strategies to implementing this approach to care. Participants in the intervention group were asked the following questions:

1. Tell me about your experiences using the iBall device?
2. What aspects of iBall were the most useful or helpful?
3. Which aspects of iBall were least useful or helpful?
4. Are there any changes you would make to the iBall device or app?
5. If you had to explain iBall to a friend, what would you say?
6. Would you recommend it to a friend? Why or why not?
7. Do you plan on continuing to use iBall at this time? Why or why not?
8. Would you consider using iBall again in the future?

**Table 4:** Description of implementation outcomes.

Outcome	Measures	Mode of analysis
Acceptability: A willingness to receive the offered intervention	Enrollment rate; attrition/retention rate; engagement/adherence rate	Research log: enrollment, follow-up and engagement tracking; analytics data; qualitative data
Feasibility: The capability to carry out intervention activities	Training of the interventionists; delivery of the program; outcome capture; perceptions of barriers and facilitators	Research log: enrollment, follow-up and engagement tracking; analytics data; qualitative data

Content analysis, following interpretive qualitative description, of participants’ responses was used to analyze the qualitative data. The investigators systematically reviewed all transcripts and inductively generated a list of codes by hand. The codes were grouped into categories and then collapsed further into broader themes [44].

## Results

A total of 23 participants were enrolled in the study. Of them, 13 were randomized to the iBall intervention group and 10 to the PFMT instruction group. Baseline characteristics were similar for both groups (Table 1).

Regarding implementation outcomes, which is the primary focus of this study, we found that, in its current form, the mHealth solution was not found to be superior to basic PFMT instruction alone (Table 5). A number of technical and logistical factors were found to hinder both the acceptability and feasibility of the mHealth intervention studied. A total of 15 categories emerged out of the qualitative analysis (Table 5), which were collapsed into 3 broader themes: (1) iBall represents an acceptable concept to support PFMT; (2) the steps involved in using iBall hinder the acceptability; (3) technology issues of this iBall are many but can be overcome.

There was no statistically significant difference between the groups for change scores (ie, 16-week score minus baseline score) for any measure (Table 6). The predetermined clinically relevant difference in PERFECT score of 20% was not achieved. Both the intervention and the standard care groups showed improvement on all outcome measures: PERFECT criteria, UDI-6, and the IIQ-7 at 16 weeks compared with baseline (Table 5). The UDI-6 score was the only outcome that achieved statistical significance in both groups.

**Table 5:** Pre- and postintervention measurements within group results.

Measurements		iBall (n=13)							Control (n=10)				
		Pre (SD)	Post (SD)	*P* value		95% CI		Pre (SD)		Post (SD)	*P* value	95% CI	
UDI-6^a,b^		18.9 (11.5)	7.3 (5.9)	.009		—^c^		25.4 (15.9)		4.6 (6.0)	.004	—	
IIQ-7^b,d^		8.1 (15.6)	3.7 (5.6)	1.00		—		7.4 (11.4)		3.2 (8.4)	.36	—	
**PERFECT score^e^**													
	Power <3/5	2 (1.3)	0 (1.5)	.27		0.03-5.25		2 (0.7)		1 (0.7)	.24	0.02-4.91	
	Endurance <6/10	2 (2.7)	0 (1.9)	.27		0.03-5.25		0 (0.7)		0 (1.4)	>.99	0.05-20.83	
	Repetitions <6/10	7 (3.1)	2 (3.7)	.07		0.01-1.32		3 (2.8)		3 (2.3)	.49	2.32-6.08	
	Fast <6/10	5 (2.7)	7 (2.9)	.051		0.02-6.35		3 (4.0)		2 (2.0)	.53	0.04-5.58	
	Coordination: Yes	6	6	.33		0.05-2.77		1		4	.06	0.04-1.52	
	Timing: Yes	6	6	1.00		0.17-6.00		1		4	.20	0.01-2.82	

^a^UDI: Urogenital Distress Inventory.

^b^*P* value calculated through the Mann-Whitney *U* test.

^c^Not applicable.

^d^IIQ: Incontinence Impact Questionnaire.

^e^iBall and control PERFECT scores are mean difference values. *P* values are calculated through Fisher exact test.

**Table 6:** Postintervention measurements results between the iBall and the control groups.

Measurements		iBall (n=13), mean (SD)	Control (n=10), mean (SD)	*P* value	95% CI
UDI-6^a,b^		7.3 (5.9)	4.6 (6.0)	.28	−8.29 to 2.89
IIQ-7^b,c^		3.7 (5.6)	3.2 (8.4)	.50	−7.21 to 6.21
**PERFECT score^d^**					
	Power>1	2 (0.7)	4 (1.3)	.09	0.06 to 3.17
	Endurance>2	6 (0.7)	6 (2.7)	.60	1.41 to 4.53
	Repetitions>2	5 (2.8)	3 (3.1)	>.99	0.32 to 11.8
	Fast>2	7 (4.0)	4 (2.7)	>.99	0.45 to 15.3
	Coordination: Yes	3	2	>.99	1.95 to 11.5
	Timing: Yes	1	2	.24	0.03 to 5.88

^a^UDI: Urogenital Distress Inventory.

^b^*P* value calculated through the Mann-Whitney *U* test.

^c^IIQ: Incontinence Impact Questionnaire.

^d^iBall and control PERFECT scores are mean difference values. *P* values are calculated through Fisher exact test.

## Discussion

### Principal Findings

The results of this randomized controlled pilot study do not support the use of the studied mHealth device in its current form when compared with PFMT instruction alone using the chosen quantitative measures. Although most participants indicated that the concept of the mHealth solution has potential, technical difficulties and a cumbersome setup were the primary themes that emerged impeding the intervention’s acceptability. Only 2 of the 11 participants would recommend the mHealth solution to a friend, although 7 would consider recommending with modifications. Only 2 participants would consider using it again in the future (Table 7). Analytics data combined with interview data highlighted the lack of adherence to the intervention protocol. Technical issues related to the mHealth solution function hampered use. Consequently, we were unable to demonstrate increased motivation and adherence when using this mHealth solution.

Although there was improvement in both groups, there was no statistically significant difference between groups. The development of pelvic floor dysfunction involves a complex process involving a multifactorial etiology manifesting in the perinatal period [45]. We found that both groups achieved satisfactory pelvic floor health at the end of the 16-week intervention period. Level 1a evidence recommends the commencement of proper pelvic floor exercises in the early postpartum period, confirmed through a digital vaginal examination [21]. We implemented this recommendation across both control and intervention groups and both groups appeared to benefit.

The lack of implementation and adherence to PFMT protocols in the early postpartum period translates to a large proportion of women (92%) continuing to have urinary incontinence 5 years postpartum if their incontinence was not resolved by 12 months postpartum [46]. Furthermore, 44.6% of women have been found to have incontinence 5 to 7 years postpartum [47]. These statistics are problematic considering that only a small proportion of women seek medical care for incontinence [48]. Our study results highlight the potential opportunity for enhanced pelvic floor muscle restoration and fitness development through more consistent implementation of postpartum PFMT.

Although the efficacy of PFMT is clearly established, the effect of adjunctive approaches is yet to demonstrate additional benefit. The International Consultation on Urinary Incontinence, a rigorous international committee-led review and analysis of the most up-to-date literature concluded that adjunctive pelvic floor muscle therapies such as biofeedback and electrical stimulation were not superior to supervised PFMT [49]. Many mHealth devices are available for use, and although they are designed to overcome some of the barriers inherent in traditional PFMT adjunctive technologies, few have been appropriately studied. A recent randomized controlled pilot study conducted to compare the Vibrance Kegel Device with a standard PFMT program suggested an added benefit to this mHealth solution [50].

The growing appeal of mobile solutions for health promotion and health care delivery can be attributed to the accessibility of the technology and the level of personalization that the technology enables [51]. Studies have shown that mHealth interventions have the potential to support successful management of chronic health conditions and associated health behavior change through (1) improving patient self-monitoring and management [52,53], (2) informing health care professionals of patients’ health status [54,55], and (3) tailoring care and education to patient needs [56-58].

**Table 7:** Qualitative findings.

Perspective	Agreement, n (%)	Supporting quotes
The concept of a device to help rehabilitation of your pelvic floor through biofeedback is good	10 (73)	“As a busy mom I find I am too busy to be going to appointments, so this allowed me to get the help I needed without going to appointments” (1001); “The concept is really good, it’s just that with a baby and a toddler time was my barrier” (1015); “I mean the premise of having a game to strengthen the pelvic floor made it intriguing” (1005)
The biofeedback was helpful	4 (31)	“When it was working it was helpful to have the feedback; I liked knowing how the pelvic floor was working” (1023); “I liked how on the strengthening aspect graded the strength of the contraction, that was cool visual feedback” (1005)
The biofeedback was not helpful (inconsistent/inaccurate)	8 (61)	“It didn’t’ reliably work so that really did not justify the effort in using it. It is not motivating using a device that is unreliable” (1002); “I was trying to squeeze as hard as I could and it just was not registering (1017)
Tracking my progress was a helpful feature	2 (15)	“Seeing your score and being able to keep track of your score so that you were working towards something was motivating. The other part was being able to see other peoples scores, that helped to give you a sense of where you ranked in comparison to other people. That was motivating too or at least made it more enticing to want to play more” (1013)
Tracking my progress was not helpful (inconsistent/inaccurate)	7 (54)	“I found it really difficult to use it properly, some of the time it would say it was connected but then none of my resulted recorded” (1023); “I did find out that the results were not getting sent in for whatever reason so this was off putting and really made me not want to use it” (1011)
Instructions were easy to follow and the purpose, clear	3 (23)	“it was pretty self-explanatory and I felt like the instructions I was given here at the beginning of the study were really clear and straight forward” (1001)
Instructions were not straight forward and the purpose, unclear	7 (54)	“It was a confusing because with the games there were no instructions, it took me multiple attempts to try to figure out what I was doing” (1005); “The instructions on the game could have been more clear, I never knew what the goal was or how long I should be playing for” (1017).
The device and app motivated me to do pelvic floor exercises (facilitator)	4 (31)	“In particular, I found the strength game really motivating” (1011); “I tried different games; the games were interesting they were like video games” (1010)
The device and app made it more difficult to do pelvic floor exercises (barrier)	8 (62)	“The other thing I realized though is that when you have a baby it is a lot harder to use a device than it is to just do the kegels that were taught to you on your own” (1002); “The hassle of using the whole device was an issue, it was not as easy as just doing the [pelvic floor] exercises (1008); “The main barriers to using it was finding time to use it; needing privacy; and the cumbersome process to set it up” (1013)
Technical difficulties with the mHealth solution were an issue	6 (69)	“When I was trying to play the games I couldn’t get any type of a score and it was frustrating because I didn’t know if it was just a problem with the app or device or if I really was not getting any engagement of my pelvic floor” (1002); “The accuracy of how the device communicates with the definitely needs improvement” (1023)
The setup of the device and app was cumbersome (not *new mom* friendly)	8 (62)	“The fact that you have to set it up and lie down, get lubricant in order to use it – so set up and clean up doesn’t mix well with the life of a busy mom who if constantly interrupted” (1015); “I was excited about it at first, but because it was too much work, a real hassle to get it set up, I didn’t use it much” (1010)
The device was comfortable	5 (38)	“It was really easy to insert which was nice” (1011); “I found it comfortable and relatively user-friendly’ (1015)
The device was uncomfortable	8 (62)	“I really haven’t been using it – because it is big and frankly the idea of inserting it is not appealing, it took me 10 minutes to insert it” (1012); “I would make it [the device] more compact – it is big and not comfortable” (1008)
Optional positioning of the device was an issue	5 (38)	“but knowing the position of it – sometimes I wasn’t sure if it was inserted too deep or too superficial” (1005); “Also, I did find slightly changing the position of the [device] really changed what the feedback indicated” (1023)
The mHealth solution was helpful when combined with a pelvic floor examination	4 (32)	“It is not useful to have this without having the assessment and some discussion with an expert’ (1017); “I don’t think a device like this would have much values at all if you didn’t also at least have some type of follow up in person” (1013)
Instruction from the practitioner was more helpful than feedback from the device and app	9 (69)	“When I did the initial exam I found that really helpful here because I was never assessed like that before but I think I would have benefited from another appointment rather than just using the device” (1010); “I feel like I need proper pelvic floor physiotherapy as I feel like everything is too tight. so, I think that is really what I need, not a device like this” (1012)

The iBall is a dynamic and interactive mHealth solution that has the capacity to enhance PFMT. With the capacity to provide biofeedback, record data, and track progress, iBall has the potential to increase motivation and adherence. Furthermore, the iBall device and smartphone app have the capability of remote storage of usage data for record keeping and additional analysis. The Web-based data feature is designed for further gamification apps. Owing to research ethics app constrains (Hamilton integrated Research Ethics Board has concerns regarding data access), the Web-based interaction features were disabled in this study. Nonetheless, based on experiences from gamification of wearable fitness devices [59], we foresee that such an interactive design feature will generally improve user motivation and adherence.

Our study has highlighted several aspects that are important for mHealth solutions to be acceptable and feasible for pelvic floor rehabilitation in the early postpartum period. Convenience, user friendliness, reliable technology, accurate biofeedback, and user progress tracking were identified as being important features (Table 7). Effective mHealth solutions require consideration of the understanding and capabilities of end users (patients and practitioners) to use the technology [60].

End user engagement throughout the design and development processes help ensure that mHealth solutions are acceptable and feasible by fitting within the end user’s context [61]. Our study results emphasize the need of end user input in the design of mHealth solutions for pelvic floor muscle function/dysfunction. Acceptability and feasibility need to be established before testing the efficacy of potential pelvic floor rehabilitation mHealth solutions in fully powered randomized controlled trials (RCTs).

A significant limitation of this study is the small sample size. We had challenges with both recruitment and follow-up. Both contributed to poor feasibility. The small sample size confers caution to interpreting the statistical findings. As we did not conduct a priori sample size calculations, the results should be considered exploratory. We acknowledge possible ceiling effects related to the outcome measures. The low burden of pelvic floor dysfunction at baseline and limitations related to the UDI-6 and IIQ-7 in the postpartum period resulted in poor discriminatory powers of the measurements. The UDI-6 and IIQ-7 are well-established psychometrically sound self-report questionnaires that were developed for symptomatic patients and not healthy women in the early postpartum period. A recent review of 33 urogynecology questionnaires confirmed that none target postpartum women and that tools specific to this population are needed [62].

This pilot trial was designed to establish feasibility and identify issues for such mHealth technologies to be used in future clinical applications. Our results have led to the identification of several issues in the current device technologies that prevent it from being used in a large-scale trial and potential future clinical use. We also identified potential solutions that were mostly related to redesigning the device for clinical therapeutic use rather than recreational applications. Such changes are easy to implement with little technological barriers. For a future clinical trial, we expect a minor redesigning of the hardware, a major revision of the software (ie, the iBall app), and adjustments to the intervention protocol to reduce loss to follow-up and potentially improve adherence.

### Conclusions

This pilot study has demonstrated that an mHealth solution may be useful in supporting PFMT. The concept of the mHealth solution was acceptable. However, several usability issues in hardware and software hindered its feasibility and acceptance as a rehabilitation tool by the participants. The results from this study affirm the potential for mHealth solutions to support PFMT in the early postpartum period, particularly with redesigning that allows for enhanced technical literacy and accurate biofeedback. We also affirmed the benefit of recommended PFMT alone, which recommends instruction of correct pelvic floor exercises through digital palpation early postpartum. Established acceptability and feasibility parameters are needed before testing potential pelvic floor rehabilitation mHealth solutions in future in fully powered RCTs to determine efficacy of this modality.

## Abbreviations

IIQ: Incontinence Impact Questionnaire
mHealth: mobile health
PFMT: pelvic floor muscle training
RCT: randomized controlled trial
UDI: Urogenital Distress Inventory

## Appendix

Multimedia Appendix 1 CONSORT‐EHEALTH checklist (V 1.6.2).
