# Supplementary material for: Exploring the Impact of a Mobile Health Solution for Postpartum Pelvic Floor Muscle Training: Pilot Randomized Controlled Feasibility Study
Source: JMIR Mhealth Uhealth. 2019 Jul 11;7(7):e12587. doi: 10.2196/12587 (PMC6657451; doi:10.2196/12587)
Supplement: Multimedia Appendix 1 [file mhealth_v7i7e12587_app1.pdf]

**CONSORT-EHEALTH Checklist V1.6.2 Report**	**Manuscript Number**	12587
**(based on CONSORT-EHEALTH V1.6), available at [http://tinyurl.com/consort-ehealth-v1-6].**		
**Date completed**		
7/9/2019 20:21:31		
**by**		
Sinead		
Exploring the Impact of a Mobile Health Solution for Postpartum Pelvic Floor Muscle Training: A Pilot Randomized Controlled Feasibility Study		
TITLE		
**1a-i) Identify the mode of delivery in the title**		
Exploring the Impact of a "Mobile Health Solution" for Postpartum Pelvic Floor Muscle Training: A Pilot Randomized Controlled Feasibility Study		
**1a-ii) Non-web-based components or important co-interventions in title**		
Exploring the Impact of a "Mobile Health Solution" for Postpartum Pelvic Floor Muscle Training: A Pilot Randomized Controlled Feasibility Study		
**1a-iii) Primary condition or target group in the title**		
Exploring the Impact of a Mobile Health Solution for "Postpartum Pelvic Floor Muscle Training": A Pilot Randomized Controlled Feasibility Study		
ABSTRACT		
**1b-i) Key features/functionalities/components of the intervention and comparator in the METHODS section of the ABSTRACT**		
Methods: A 16-week mixed-methods pilot study was conducted to evaluate outcomes and determine aspects of acceptability and feasibility of an mHealth device. All participants received standardized examination of their pelvic floor muscles and associated instruction on the correct performance of PFMT. Those randomized to the iBall intervention received instructions on its use. Schedules for utilization of the iBall and PFMT were not prescribed, but all participants were informed of the standard established recommendation of PFMT, which includes 3 sets of 10 exercises, 3 to 4 times a week, for the duration of the intervention period. Quantitative data included the measurement of pelvic floor muscle parameters (strength, endurance, and co-ordination) following the PERFECT assessment scheme: Incontinence Impact Questionnaire scores and the Urogenital Distress Inventory (UDI-6) scores. Aspects of acceptability and feasibility were collected through one-to-one interviews. Interview transcripts were analyzed using Thorne's interpretive description approach.		
**1b-ii) Level of human involvement in the METHODS section of the ABSTRACT**		
All participants received standardized examination of their pelvic floor muscles and associated instruction on the correct performance of PFMT. Those randomized to the iBall intervention received instructions on its use. Schedules for utilization of the iBall and PFMT were not prescribed, but all participants were informed of the standard established recommendation of PFMT, which includes 3 sets of 10 exercises, 3 to 4 times a week, for the duration of the intervention period.		
**1b-iii) Open vs. closed, web-based (self-assessment) vs. face-to-face assessments in the METHODS section of the ABSTRACT**		
Assessments were in person as this is the needed gold standard to properly assess the pelvic floor. The abstract indicates this.<br>"All participants received standardized examination of their pelvic floor muscles and associated instruction on the correct performance of PFMT. Those randomized to the iBall intervention received instructions on its use. Schedules for utilization of the iBall and PFMT were not prescribed, but all participants were informed of the standard established recommendation of PFMT, which includes 3 sets of 10 exercises, 3 to 4 times a week, for the duration of the intervention period."		
**1b-iv) RESULTS section in abstract must contain use data**		

Results: A total of 23 women with a mean age of 32.2 years were randomized to an intervention group (n=13) or a control group (n=10). Both groups improved on all measures. The only statistically significant change was the UDI-6 score within both groups at 16 weeks compared with baseline. There was no statistically significant difference between the intervention group and control group on any outcomes. Most participants using the iBall 10(77%) indicated value in the concept of the mHealth solution. Technical difficulties 10(77%), a cumbersome initiation process 8(61%), and discomfort from the device 8 (61%) were reasons impeding intervention acceptability. Most participants 17(74%) indicated that the initial assessment and training was more useful than the mHealth solution, a tenet that was echoed by all control group participants.		
**1b-v) CONCLUSIONS/DISCUSSION in abstract for negative trials**		
Conclusions: Our pilot study demonstrated the potential for mHealth solution–enhanced PFMT in the early postpartum period. Usability issues in hardware and software hindered feasibility and acceptance by the participants. Our findings can inform the redesign of mHealth solutions that may be of value if acceptability and feasibility issues can be overcome.		
INTRODUCTION		
**2a-i) Problem and the type of system/solution**		
Problem:<br>"The postpartum period is a vulnerable time for the pelvic floor [1]. Dysfunction of the pelvic floor musculature is associated with urinary and fecal incontinence, pelvic organ prolapse, and lumbopelvic pain [2-8]. Prevalence data vary; however, approximately 23% of postpartum women report either urinary or fecal incontinence [63]. "<br><br>"PFMT can be effectively initiated with brief instruction through digital palpation of the pelvic floor muscles as associated feedback related to the muscle contraction completed [20]. It is established that, in the absence of providing adequate instruction and biofeedback, it is difficult for women to accurately perform pelvic floor muscle exercises [22,23]. Existing literature suggests that best practices for the prevention and management of pelvic floor dysfunction are not routinely implemented [24,25]. There is no universally accepted protocol for the initiation of PFMT in the postpartum period. This represents a research-practice gap."<br><br>Solution:<br><br>"A proactive approach to pelvic health is needed as are strategies to overcome barriers contributing to this research-practice gap. Mobile health (mHealth) solutions may provide strategies to close the gap. mHealth solutions are defined by the World Health Organization [26] as the “medical and public health practice supported by mobile devices, such as mobile phones, patient monitoring devices, personal digital assistants, and other wireless devices” [26]."<br><br>"This pilot randomized controlled study aimed to evaluate the feasibility and acceptability of an mHealth solution (iBall) as a rehabilitation tool to support PFMT efforts in the early postpartum period. The iBall is a device that, upon insertion into the vaginal canal, can detect the strength and muscular endurance of pelvic floor muscles. The results are displayed in the accompanying mobile app that incorporates various training routines and gaming options. It aims to encourage both adherence to and improvement of outcomes of PFMT. Specifically, we sought to assess the feasibility, acceptability, and effectiveness of outcomes of an mHealth device (iBall) compared with PFMT instruction alone in the early postpartum period."		
**2a-ii) Scientific background, rationale: What is known about the (type of) system**		
"The current market for mHealth technologies is growing, including applications marketed for pelvic floor rehabilitation and fitness [53-56]. These mHealth technologies may represent innovative modalities to support pelvic floor muscle fitness. Claims and proposed benefits of these mHealth solutions, compared with standard care, are based on concepts and have not been substantiated by scientific investigation. Overall, the body of evidence on the effectiveness of mHealth technology is, in general, weak [27]."		
**Does your paper address CONSORT subitem 2b?**		
Yes - purpose and aim clearly explained.		
METHODS		

**3a) CONSORT: Description of trial design (such as parallel, factorial) including allocation ratio**		
"A 16-week mixed-methods pilot study was conducted to evaluate outcomes and<br>This study was done in a 1:1 allocation ratio using random number assignments. Allocations were placed in sealed envelopes that were opened after the initial physical assessment at the time of randomization. Women having a vaginal or cesarean birth within 21 weeks of delivery were included. To maximize generalizability, the exclusions were made based on the individuals' inability to understand and read English and direction from their caregivers to not insert anything into their vagina."<br><br>"An initial phone screening was conducted by a research assistant. Interested and eligible participants then met with a research assistant for further discussion and to obtain informed consent. The start time of the intervention took place in the early postpartum period, primarily during the fourth trimester (from week 6 postpartum to week 13 postpartum)."		
**3b) CONSORT: Important changes to methods after trial commencement (such as eligibility criteria), with reasons**		
None		
**3b-i) Bug fixes, Downtimes, Content Changes**		
"We were evaluating these aspects so the findings reflect these items.<br>Most participants using the iBall 10(77%) indicated value in the concept of the mHealth solution. Technical difficulties 10(77%), a cumbersome initiation process 8(61%), and discomfort from the device 8(61%) were reasons impeding intervention acceptability. Most participants 17(74%) indicated that the initial assessment and training was more useful than the mHealth solution, a tenet that was echoed by all control group participants."		
**4a) CONSORT: Eligibility criteria for participants**		
Yes - in methods.<br><br>"Women in the third trimester of pregnancy, attending local midwifery practices, were invited to participate in the study."		
**4a-i) Computer / Internet literacy**		
No		
**4a-ii) Open vs. closed, web-based vs. face-to-face assessments:**		
In person assessments.<br><br>Following assessment, subjects received their randomization allocation. Subjects randomized to the iBall group received additional instruction and training from the research assistant. Follow-up assessments were completed by the same assessors (SD and DF). A consistent process for assessment procedures including cueing during the pelvic floor muscle testing and instruction for the performance of pelvic floor exercises was established. All participants received instruction for the correct performance of pelvic floor muscle exercises via digital palpation. Schedules for utilization of the iBall and PFMT were not prescribed, but all participants were informed of the standard established recommendation of PFMT, which includes 3 sets of 10 exercises, 3 to 4 times a week, for duration of the intervention period [28]. Neither assessor was at any time, during the conduct of the study, aware of which group a subject was allocated.		
**4a-iii) Information giving during recruitment**		
Posters were used to facilitate recruitment. All initial study procedures took place in the early postpartum period (within the fourth trimester, the first 13 weeks postpartum). Following initial screening and confirmation of eligibility, participants were prospectively randomized to an mHealth-assisted PFMT intervention or PFMT instruction only (Figure 1). The study was approved by the Hamilton Integrated Research and Ethics Board.		
**4b) CONSORT: Settings and locations where the data were collected**		
Yes		
**4b-i) Report if outcomes were (self-)assessed through online questionnaires**		
no online questionnaires used		

**4b-ii) Report how institutional affiliations are displayed**		
In association with authors only.		
**5) CONSORT: Describe the interventions for each group with sufficient details to allow replication, including how and when they were actually administered**		
**5-i) Mention names, credential, affiliations of the developers, sponsors, and owners**		
The iBall (ChunShuiTang Co Ltd) is an interactive mHealth device designed to facilitate PFMT. The iBall device is a US Federal Communications Commission–certified [38] and European Conformity–marked [39,40] device that, upon insertion into the vaginal canal, can detect the strength (power generation) and associated muscular endurance of pelvic floor muscles. Currently, iBall has been approved for consumer use in Europe and China. This information detected from the sensors within the device are transmitted via Bluetooth to a smartphone app that guides users in various exercise programs. The device is shown in Figure 1. It comprises 2 spherical compartments and a Bluetooth antenna for wireless communication with the smartphone. The whole device including the antenna is encapsulated in waterproof medical grade silicone casing. The battery and charging circuit are in the top spherical compartment with a waterproof charging port in the silicone encapsulation. The sensor and biofeedback motors are in the bottom compartment, which also contains a power button under the silicone casing. The user can press and hold the power button to turn on/off the device. The antenna also serves as a handle to push in and pull out the device from the vaginal canal. In this study, each participant in the intervention group will receive an iBall device along with an already paired smartphone (iPhone 5e) with the mobile app installed and tested with the device. Both the iBall device and the smartphone were originally fully charged, and power will last a few days depending on usage level.<br><br>These quantitative results are saved with a time stamp in the mobile app. The data can also be automatically uploaded to an encrypted cloud storage server through an opt in option. In this study, the participants have consented to upload their usage data for the study with the uploading feature enabled. Each user's study smartphone app has already been configured with a unique randomly generated user account and log-in credential. Only the research team has a securely saved lookup table linking the user account and the identity of the participants. The cloud-stored data can be downloaded as structured data files (in American Standard Code for Information Interchange/ASCII format) for further analysis. The original consumer product has a Web-based profile function allowing the users to access their data on the cloud server and interact (by users' choice) with other users in a Web-based community. This feature is only available in Chinese and was permanently disabled in the clinical study version of the smartphone app. As a result, although the device and app have the capability of Web access to their usage data and interacting with other users, such functionalities were disabled in devices used in this study. The iBall device is currently marked as a consumer electronics gaming device. Some quantitative results (eg, power or contracting force) are not calibrated for absolute pressure measurements. The results of metrics obtained from the devices are not included in this study.		
**5-ii) Describe the history/development process**		
Not applicable.		
**5-iii) Revisions and updating**		

Discussed in the implications section.<br><br>The iBall is a dynamic and interactive mHealth solution that has the capacity to enhance PFMT. With the capacity to provide biofeedback, record data, and track progress, iBall has the potential to increase motivation and adherence. Furthermore, the iBall device and smartphone app have the capability of remote storage of usage data for record keeping and additional analysis. The Web-based data feature is designed for further gamification apps. Owing to research ethics app constrains (Hamilton integrated Research Ethics Board/HiREB has concerns regarding data access), the Web-based interaction features were disabled in this study. Nonetheless, based on experiences from gamification of wearable fitness devices [62], we foresee that such an interactive design feature will generally improve user motivation and adherence.<br>Our study has highlighted several aspects that are important for mHealth solutions to be acceptable and feasible for pelvic floor rehabilitation in the early postpartum period. Convenience, user friendliness, reliable technology, accurate biofeedback, and user progress tracking were identified as being important features (Table 7). Effective mHealth solutions require consideration of the understanding and capabilities of end users (patients and practitioners) to use the technology [49].<br>End user engagement throughout the design and development processes help ensure that mHealth solutions are acceptable and feasible by fitting within the end user's context [50]. Our study results emphasize the need of end user input in the design of mHealth solutions for pelvic floor muscle function/dysfunction. Acceptability and feasibility need to be established before testing the efficacy of potential pelvic floor rehabilitation mHealth solutions in fully powered randomized controlled trials (RCTs).		
**5-iv) Quality assurance methods**		
Limitations explicated clearly and attention to related to qualitative and quantitative methods assurance measures attended to.<br><br>A significant limitation of this study is the small sample size. We had challenges with both recruitment and follow-up. Both contributed to poor feasibility. The small sample size confers caution to interpreting the statistical findings. As we did not conduct a priori sample size calculations, the results should be considered exploratory. We acknowledge possible ceiling effects related to the outcome measures. The low burden of pelvic floor dysfunction at baseline and limitations related to the UDI-6 and IIQ-7 in the postpartum period resulted in poor discriminatory powers of the measurements. The UDI-6 and IIQ-7 are well-established psychometrically sound self-report questionnaires that were developed for symptomatic patients and not healthy women in the early postpartum period. A recent review of 33 urogynecology questionnaires confirmed that none target postpartum women and that tools specific to this population are needed [51].		
**5-v) Ensure replicability by publishing the source code, and/or providing screenshots/screen-capture video, and/or providing flowcharts of the algorithms used**		
All provided as figures.		
**5-vi) Digital preservation**		
Not addressed.		
**5-vii) Access**		
Participants randomized to the intervention group received additional instruction on how to use the mHealth solution (iBall) by a research assistant. Participants were shown how to use the device and were familiarized with the associated app on the associated smartphone. Maintenance was informed as per manufacturers recommendations, which involved washing the device with warm water and a natural soap and storing it in the case it came in. Participants then used the device according to their own schedule (eg, when to exercise, frequency, and how long). The PFMT with iBall occurs within the context of playing a variety of games. Participants in the intervention group received a booster session, midway through the intervention period. This booster session consisted of an email sent by a research assistant reminding participants of features of the iBall and benefits of postpartum pelvic floor muscle exercise as a means of self-management support. The use of booster sessions to facilitate self-management support has been shown to be a useful procedure in rehabilitation interventions [29].		
**5-viii) Mode of delivery, features/functionalities/components of the intervention and comparator, and the theoretical framework**		

The iBall (ChunShuiTang Co Ltd) is an interactive mHealth device designed to facilitate PFMT. The iBall device is a US Federal Communications Commission–certified [38] and European Conformity–marked [39,40] device that, upon insertion into the vaginal canal, can detect the strength (power generation) and associated muscular endurance of pelvic floor muscles. Currently, iBall has been approved for consumer use in Europe and China. This information detected from the sensors within the device are transmitted via Bluetooth to a smartphone app that guides users in various exercise programs. The device is shown in Figure 1. It comprises 2 spherical compartments and a Bluetooth antenna for wireless communication with the smartphone. The whole device including the antenna is encapsulated in waterproof medical grade silicone casing. The battery and charging circuit are in the top spherical compartment with a waterproof charging port in the silicone encapsulation. The sensor and biofeedback motors are in the bottom compartment, which also contains a power button under the silicone casing. The user can press and hold the power button to turn on/off the device. The antenna also serves as a handle to push in and pull out the device from the vaginal canal. In this study, each participant in the intervention group will receive an iBall device along with an already paired smartphone (iPhone 5e) with the mobile app installed and tested with the device. Both the iBall device and the smartphone were originally fully charged, and power will last a few days depending on usage level. They both require periodic charging by the participants at home during the study period. Once inserted, the user has several interactive games or activities to choose from on the corresponding app on their smartphone (Figure 2). Their progress can also be tracked, both as a score in the game and as a measure of power, endurance, repetitions, and contracting force (Figure 2).		
**5-ix) Describe use parameters**		
Following assessment, subjects received their randomization allocation. Subjects randomized to the iBall group received additional instruction and training from the research assistant. Follow-up assessments were completed by the same assessors (SD and DF). A consistent process for assessment procedures including cueing during the pelvic floor muscle testing and instruction for the performance of pelvic floor exercises was established. All participants received instruction for the correct performance of pelvic floor muscle exercises via digital palpation. Schedules for utilization of the iBall and PFMT were not prescribed, but all participants were informed of the standard established recommendation of PFMT, which includes 3 sets of 10 exercises, 3 to 4 times a week, for duration of the intervention period [28].		
**5-x) Clarify the level of human involvement**		
Following assessment, subjects received their randomization allocation. Subjects randomized to the iBall group received additional instruction and training from the research assistant. Follow-up assessments were completed by the same assessors (SD and DF). A consistent process for assessment procedures including cueing during the pelvic floor muscle testing and instruction for the performance of pelvic floor exercises was established. All participants received instruction for the correct performance of pelvic floor muscle exercises via digital palpation. Schedules for utilization of the iBall and PFMT were not prescribed, but all participants were informed of the standard established recommendation of PFMT, which includes 3 sets of 10 exercises, 3 to 4 times a week, for duration of the intervention period [28]. Neither assessor was at any time, during the conduct of the study, aware of which group a subject was allocated.		
**5-xi) Report any prompts/reminders used**		
Booster session used.<br><br>"Participants randomized to the intervention group received additional instruction on how to use the mHealth solution (iBall) by a research assistant. Participants were shown how to use the device and were familiarized with the associated app on the associated smartphone. Maintenance was informed as per manufacturers recommendations, which involved washing the device with warm water and a natural soap and storing it in the case it came in. Participants then used the device according to their own schedule (eg, when to exercise, frequency, and how long). The PFMT with iBall occurs within the context of playing a variety of games. Participants in the intervention group received a booster session, midway through the intervention period. This booster session consisted of an email sent by a research assistant reminding participants of features of the iBall and benefits of postpartum pelvic floor muscle exercise as a means of self-management support. The use of booster sessions to facilitate self-management support has been shown to be a useful procedure in rehabilitation interventions [29]."		
**5-xii) Describe any co-interventions (incl. training/support)**		
Participants were instructed on the use of iball prior to the study.		
**6a) CONSORT: Completely defined pre-specified primary and secondary outcome measures, including how and when they were assessed**		

The assessment included completion of 2 self-report outcome measures and a digital pelvic floor examination, inclusive of initiating PFMT by one of the 2 assessors. The 2 validated self-report measures that were administered were the Urogenital Distress Inventory (UDI-6) [30] and the Incontinence Impact Questionnaire (IIQ-7), which are shown in Table 2. The pelvic floor muscle examination followed the PERFECT scheme [8]. Clinically relevant changes in the PERFECT score was predetermined as an improvement of 20% based on previously published minimal clinical improvement thresholds.		
**6a-i) Online questionnaires: describe if they were validated for online use and apply CHERRIES items to describe how the questionnaires were designed/deployed**		
no online questionnaires used.		
**6a-ii) Describe whether and how “use” (including intensity of use/dosage) was defined/measured/monitored**		
self-report through one-to-one interviews and corroborated through analytic data.		
**6a-iii) Describe whether, how, and when qualitative feedback from participants was obtained**		
Mixed methods pilot feasibility study - one to one interview data represented a large part of the study data.		
**6b) CONSORT: Any changes to trial outcomes after the trial commenced, with reasons**		
Yes		
**7a) CONSORT: How sample size was determined**		
**7a-i) Describe whether and how expected attrition was taken into account when calculating the sample size**		
Not important given feasibility study.		
**7b) CONSORT: When applicable, explanation of any interim analyses and stopping guidelines**		
The assessment included completion of 2 self-report outcome measures and a digital pelvic floor examination, inclusive of initiating PFMT by one of the 2 assessors. The 2 validated self-report measures that were administered were the Urogenital Distress Inventory (UDI-6) [30] and the Incontinence Impact Questionnaire (IIQ-7), which are shown in Table 2. The pelvic floor muscle examination followed the PERFECT scheme [8]. Clinically relevant changes in the PERFECT score was predetermined as an improvement of 20% based on previously published minimal clinical improvement thresholds.		
**8a) CONSORT: Method used to generate the random allocation sequence**		
computer generated.		
**8b) CONSORT: Type of randomisation; details of any restriction (such as blocking and block size)**		
computer generated - no restrictions.		
**9) CONSORT: Mechanism used to implement the random allocation sequence (such as sequentially numbered containers), describing any steps taken to conceal the sequence until interventions were assigned**		
Computer generated - assessors blind.		
**10) CONSORT: Who generated the random allocation sequence, who enrolled participants, and who assigned participants to interventions**		
Research assistant		
**11a) CONSORT: Blinding - If done, who was blinded after assignment to interventions (for example, participants, care providers, those assessing outcomes) and how**		
**11a-i) Specify who was blinded, and who wasn’t**		
Researchers and assessor were blind.		
**11a-ii) Discuss e.g., whether participants knew which intervention was the “intervention of interest” and which one was the “comparator”**		
Participants knew what group they were in.		
**11b) CONSORT: If relevant, description of the similarity of interventions**		
Both involved education related to pelvic floor muscle training.		
**12a) CONSORT: Statistical methods used to compare groups for primary and secondary outcomes**		
There was no statistically significant difference between the groups for change scores (ie, 16-week score minus baseline score) for any measure (Table 5). The predetermined clinically relevant difference in PERFECT score of 20% was not achieved. Both the intervention and the standard care groups showed improvement on all outcome measures: PERFECT criteria, UDI-6, and the IIQ-7 at 16 weeks compared with baseline (Table 6). The UDI-6 score was the only outcome that achieved statistical significance in both groups.		

**12a-i) Imputation techniques to deal with attrition / missing values**		
Analysis completed for full data sets.		
**12b) CONSORT: Methods for additional analyses, such as subgroup analyses and adjusted analyses**		
Yes		
RESULTS		
**13a) CONSORT: For each group, the numbers of participants who were randomly assigned, received intended treatment, and were analysed for the primary outcome**		
Yes		
**13b) CONSORT: For each group, losses and exclusions after randomisation, together with reasons**		
Yes		
**13b-i) Attrition diagram**		
Yes - flow chart provided		
**14a) CONSORT: Dates defining the periods of recruitment and follow-up**		
No dates indicated.		
**14a-i) Indicate if critical “secular events” fell into the study period**		
No		
**14b) CONSORT: Why the trial ended or was stopped (early)**		
It was not ended early.		
**15) CONSORT: A table showing baseline demographic and clinical characteristics for each group**		
Yes		
**15-i) Report demographics associated with digital divide issues**		
N/A		
**16a) CONSORT: For each group, number of participants (denominator) included in each analysis and whether the analysis was by original assigned groups**		
**16-i) Report multiple “denominators” and provide definitions**		
Yes		
**16-ii) Primary analysis should be intent-to-treat**		
Yes - but this was a pilot feasibility study.		
**17a) CONSORT: For each primary and secondary outcome, results for each group, and the estimated effect size and its precision (such as 95% confidence interval)**		
Confidence intervals provided.		
**17a-i) Presentation of process outcomes such as metrics of use and intensity of use**		
Discussed in qualitative findings.		
**17b) CONSORT: For binary outcomes, presentation of both absolute and relative effect sizes is recommended**		
Yes - n and % indicated.		
**18) CONSORT: Results of any other analyses performed, including subgroup analyses and adjusted analyses, distinguishing pre-specified from exploratory**		
Yes		
**18-i) Subgroup analysis of comparing only users**		
N/A -		
**19) CONSORT: All important harms or unintended effects in each group**		
No harms in study - all negative impacts of the intervention captured in the qualitative findings.		
**19-i) Include privacy breaches, technical problems**		

All technical issues captured in the qualitative findings.		
**19-ii) Include qualitative feedback from participants or observations from staff/researchers**		
Large portion of the study.		
DISCUSSION		
**20) CONSORT: Trial limitations, addressing sources of potential bias, imprecision, multiplicity of analyses**		
**20-i) Typical limitations in ehealth trials**		
A significant limitation of this study is the small sample size. We had challenges with both recruitment and follow-up. Both contributed to poor feasibility. The small sample size confers caution to interpreting the statistical findings. As we did not conduct a priori sample size calculations, the results should be considered exploratory. We acknowledge possible ceiling effects related to the outcome measures. The low burden of pelvic floor dysfunction at baseline and limitations related to the UDI-6 and IIQ-7 in the postpartum period resulted in poor discriminatory powers of the measurements. The UDI-6 and IIQ-7 are well-established psychometrically sound self-report questionnaires that were developed for symptomatic patients and not healthy women in the early postpartum period. A recent review of 33 urogynecology questionnaires confirmed that none target postpartum women and that tools specific to this population are needed [51].		
**21) CONSORT: Generalisability (external validity, applicability) of the trial findings**		
**21-i) Generalizability to other populations**		
This pilot study has demonstrated that an mHealth solution may be useful in supporting PFMT. The concept of the mHealth solution was acceptable. However, several usability issues in hardware and software hindered its feasibility and acceptance as a rehabilitation tool by the participants. The results from this study affirm the potential for mHealth solutions to support PFMT in the early postpartum period, particularly with redesigning that allows for enhanced technical literacy and accurate biofeedback. We also affirmed the benefit of recommended PFMT alone, which recommends instruction of correct pelvic floor exercises through digital palpation early postpartum. Established acceptability and feasibility parameters are needed before testing potential pelvic floor rehabilitation mHealth solutions in future in fully powered RCTs to determine efficacy of this modality.		
**21-ii) Discuss if there were elements in the RCT that would be different in a routine application setting**		
Only with improvements to the current mHealth solution.		
**22) CONSORT: Interpretation consistent with results, balancing benefits and harms, and considering other relevant evidence**		
**22-i) Restate study questions and summarize the answers suggested by the data, starting with primary outcomes and process outcomes (use)**		
The results of this randomized controlled pilot study do not support the use of the studied mHealth device in its current form when compared with PFMT instruction alone using the chosen quantitative measures. Although most participants indicated that the concept of the mHealth solution has potential, technical difficulties and a cumbersome setup were the primary themes that emerged impeding the intervention’s acceptability. Only 2 of the 11 participants would recommend the mHealth solution to a friend, although 7 would consider recommending with modifications. Only 2 participants would consider using it again in the future (Table 7). Analytics data combined with interview data highlighted the lack of adherence to the intervention protocol. Technical issues related to the mHealth solution function hampered use. Consequently, we were unable to demonstrate increased motivation and adherence when using this mHealth solution.<br>Although there was improvement in both groups, there was no statistically significant difference between groups. The development of pelvic floor dysfunction involves a complex process involving a multifactorial etiology manifesting in the perinatal period [32]. We found that both groups achieved satisfactory pelvic floor health at the end of the 16-week intervention period. Level 1a evidence recommends the commencement of proper pelvic floor exercises in the early postpartum period, confirmed through a digital vaginal examination [20]. We implemented this recommendation across both control and intervention groups and both groups appeared to benefit.<br>The lack of implementation and adherence to PFMT protocols in the early postpartum period translates to a large proportion of women (92%) continuing to have urinary incontinence 5 years postpartum if their incontinence was not resolved by 12 months postpartum [33]. Furthermore, 44.6% of women have been found to have incontinence 5 to 7 years postpartum [34]. These statistics are problematic considering that only a small proportion of women seek medical care for incontinence [35]. Our study results highlight the potential opportunity for enhanced pelvic floor muscle restoration and fitness development through more consistent implementation of postpartum PFMT.		
**22-ii) Highlight unanswered new questions, suggest future research**		

The growing appeal of mobile solutions for health promotion and health care delivery can be attributed to the accessibility of the technology and the level of personalization that the technology enables [41]. Studies have shown that mHealth interventions have the potential to support successful management of chronic health conditions and associated health behavior change through (1) improving patient self-monitoring and management [42,43], (2) informing health care professionals of patients' health status [44,45], and (3) tailoring care and education to patient needs [46-48].<br><br>This pilot trial was designed to establish feasibility and identify issues for such mHealth technologies to be used in future clinical applications. Our results have led to the identification of several issues in the current device technologies that prevent it from being used in a large-scale trial and potential future clinical use. We also identified potential solutions that were mostly related to redesigning the device for clinical therapeutic use rather than recreational applications. Such changes are easy to implement with little technological barriers. For a future clinical trial, we expect a minor redesigning of the hardware, a major revision of the software (ie, the iBall app), and adjustments to the intervention protocol to reduce loss to follow-up and potentially improve adherence.		
Other information		
**23) CONSORT: Registration number and name of trial registry**		
Here is the link for trial registration details.<br>https://clinicaltrials.gov/ct2/show/NCT02865954?term=dufour&draw=2&rank=11		
**24) CONSORT: Where the full trial protocol can be accessed, if available**		
Here is the link for trial registration details.<br>https://clinicaltrials.gov/ct2/show/NCT02865954?term=dufour&draw=2&rank=11		
**25) CONSORT: Sources of funding and other support (such as supply of drugs), role of funders**		
This study is supported, in part, by the Natural Sciences and Engineering Research Council of Canada through an Undergraduate Summer Research Award to S Deng and Discover Grant (365065 QF). QF holds the Canada Research Chair in Biophotonics.		
**X26-i) Comment on ethics committee approval**		
Yes.		
**x26-ii) Outline informed consent procedures**		
Yes - participants were screened and consented.		
**X26-iii) Safety and security procedures**		
Not discussed.		
**X27-i) State the relation of the study team towards the system being evaluated**		
No conflicts of interest.		
